# **A **new 3D-printed temporal bone: ‘the SAPIENS’—specific anatomical printed-3D-model in education and new surgical simulations

**DOI:** 10.1007/s00405-024-08645-6

**Published:** 2024-04-29

**Authors:** Giannicola Iannella, Annalisa Pace, Alessandro Mucchino, Antonio Greco, Armando De Virgilio, Jerome R. Lechien, Antonino Maniaci, Salvatore Cocuzza, Tiziano Perrone, Daniela Messineo, Giuseppe Magliulo

**Affiliations:** 1 Department of ‘Organi di Senso’, University “Sapienza”, Viale dell’Università, 33, 00185 Rome, Italy; 2 Faculty of Medicine and Pharmacy, University of Mons (UMons), Mons, Belgium; 3 Department of Otolaryngology, Kore University, Enna, Italy; 4 Department of Medical, Surgical Sciences and Advanced Technologies G.F. Ingrassia, University of Catania, Catania, Italy; 5 Department of Otolaryngology, Civil Hospital of Alghero, Alghero, Italy

**Keywords:** 3D-printed, Temporal bone, Temporal bone dissection, Ear surgery, Training

## Abstract

**Purpose:**

Otology and neuro-otology surgeries pose significant challenges due to the intricate and variable anatomy of the temporal bone (TB), requiring extensive training. In the last years 3D-printed temporal bone models for otological dissection are becoming increasingly popular. In this study, we presented a new 3D-printed temporal bone model named 'SAPIENS', tailored for educational and surgical simulation purposes.

**Methods:**

The 'SAPIENS' model was a collaborative effort involving a multidisciplinary team, including radiologists, software engineers, ENT specialists, and 3D-printing experts. The development process spanned from June 2022 to October 2023 at the Department of Sense Organs, Sapienza University of Rome.

Acquisition of human temporal bone images; temporal bone rendering; 3D-printing; post-printing phase; 3D-printed temporal bone model dissection and validation.

**Results:**

The 'SAPIENS' 3D-printed temporal bone model demonstrated a high level of anatomical accuracy, resembling the human temporal bone in both middle and inner ear anatomy. The questionnaire-based assessment by five experienced ENT surgeons yielded an average total score of 49.4 ± 1.8 out of 61, indicating a model highly similar to the human TB for both anatomy and dissection. Specific areas of excellence included external contour, sigmoid sinus contour, cortical mastoidectomy simulation, and its utility as a surgical practice simulator.

**Conclusion:**

We have designed and developed a 3D model of the temporal bone that closely resembles the human temporal bone. This model enables the surgical dissection of the middle ear and mastoid with an excellent degree of similarity to the dissection performed on cadaveric temporal bones.

## Introduction

Otology and neuro-otology surgeries are particularly complex due to the challenging and variable anatomy of the temporal bone (TB) region, requiring extensive training to manage intricate surgical procedures [1–5]. The anatomical knowledge of TB structures is difficult to acquire directly from traditional two-dimensional images in textbooks and online resources [1–8]. This challenge is especially pronounced in middle ear and skull-base surgery, where operators often drill through the temporal bone to access the deep recesses of the mastoid, middle ear, labyrinth, and the skull base. In vivo, intraoperative damage to important structures such as the cochlea, facial nerve, or carotid artery may result in severe disabilities, including hearing loss, facial disfigurement, or stroke [1–11]. Therefore, to reduce patients' iatrogenic complications, preliminary exercises of TB dissection in the laboratory, using human temporal bone, are mandatory for all surgeons approaching this type of surgery [1–8]. According to Aussedat C. et al., for proper oto-surgical training, 10 human petrous bones should be dissected before starting the same procedures on real patients [12].

However, nowadays, it has become increasingly difficult for a surgeon in training to perform this type of dissection due to the lack of availability of human anatomical preparations, high costs ($500–$700 per specimen), disposal challenges, and limited equipped dissection laboratories. Moreover, biological risks and special disposal programs are often associated with the use of human anatomical specimens. Therefore, researchers have recently made continued efforts to create an accurate training environment that does not rely on cadaveric specimens [12–15].

Three-dimensional (3D) printing has developed significantly in recent years since its inception, and its applications are now widely used within different medical fields. One novel use of 3D printing in healthcare has been the development of anatomical models for surgical education and training. Different 3D-printed temporal models have been developed and described in the literature in recent years, and various studies of 3D-printed temporal bone models demonstrate significant likeness to cadaveric bone in drilling and dissection [13–30]. A 2021 clinical review by Frithioff et al. [13] reported that different 3D-printed temporal bone models have been proposed. The authors showed that, to be valid for dissection, 3D printed models must faithfully reproduce all the anatomical structures of the temporal bone and lateral skull base, be dissectible similarly to human specimens, and be easily reproducible.

In this preliminary report, we present the temporal bone dissection model designed and created by us, called 'SAPIENS' emphasizing the educational, skill improvement, and surgical simulation features of this TB model. The aim of our project was to develop a highly anatomically accurate model capable of reproducing the anatomy of the mastoid, middle ear, cochlea, and labyrinth, allowing for the dissection of the mastoid bone and the facial nerve within the temporal bone while studying their relationship with the middle ear, labyrinth, cochlea, and mastoid bone. In this preliminary study, we detail the development stages of our temporal bone dissection model and its characteristics. Additionally, impressions obtained from its dissection by young ENT surgeons were collected and reported.

## Materials and methods

The development and realization of the described 3D-temporal bone model took place from June 2022 to October 2023 at the Department of Sense Organs, Sapienza University of Rome. The project received IRB exemption from the Institutional Review Board at Sapienza University of Rome. A multidisciplinary team, including radiologists, software engineers, ENT specialists, and 3D-printing experts, collaborated on this unique project of a 3D-printed TB model. The entire research team agreed to call the final 3D-Printed Temporal Bones the 'SAPIENS' (**S**pecific **A**natomical **P**rinted 3D-model **I**n **E**ducation **N**ew dissection skills and **S**urgical simulation), based on its shared initial values of education and training program ideas that led to the development of the project itself.

## Development and fabrication of 3D-printed temporal bone model

### Acquisition of human temporal bone images

The process began with acquiring high-resolution CT scans of normal subjects to create a 3D model. CT scanning followed a routine protocol (120 kV, collimation 0.625 mm, reconstruction gap 0.2 mm, matrix 512 Å ~ 512, convolution kernel for bone, pitch 0.52, rotation time 0.8 s) generating thin layer images of 0.5 mm. Temporal bone CT images were anonymized and acquired as uncompressed Digital Imaging and Communications in Medicine (DICOM) datasets files, processed in the open-source software 3D Slicer (3D Slicer version 4.8, The Slicer Community, Boston, MA; available at www.slicer.org; accessed 27 September 2018). This ensured accurate representation after reconciling imaging artifacts and errors.

### Temporal bone rendering

To realize the 3D rendering of the temporal bone and to optimize the file for 3D printing, TB images were processed using Meshmixer software (Meshmixer 3.5, Autodesk Inc.,San Rafael, California; available at www.meshmixer.com; accessed August 2018). The software allowed reworking of all temporal bone structures, to understand and verify their correct reproducibility in the project. The software has a few options to mark the different anatomical structures. Therefore it has been used the normal setting to markup bones, through a compatible threshold with the bone profile. The facial nerve, due to CT-presented thin bone laminae and dehiscences, required manual completion. The final rendered TB model was saved in.stl format.

### 3D-printing

**Fig. 1:**
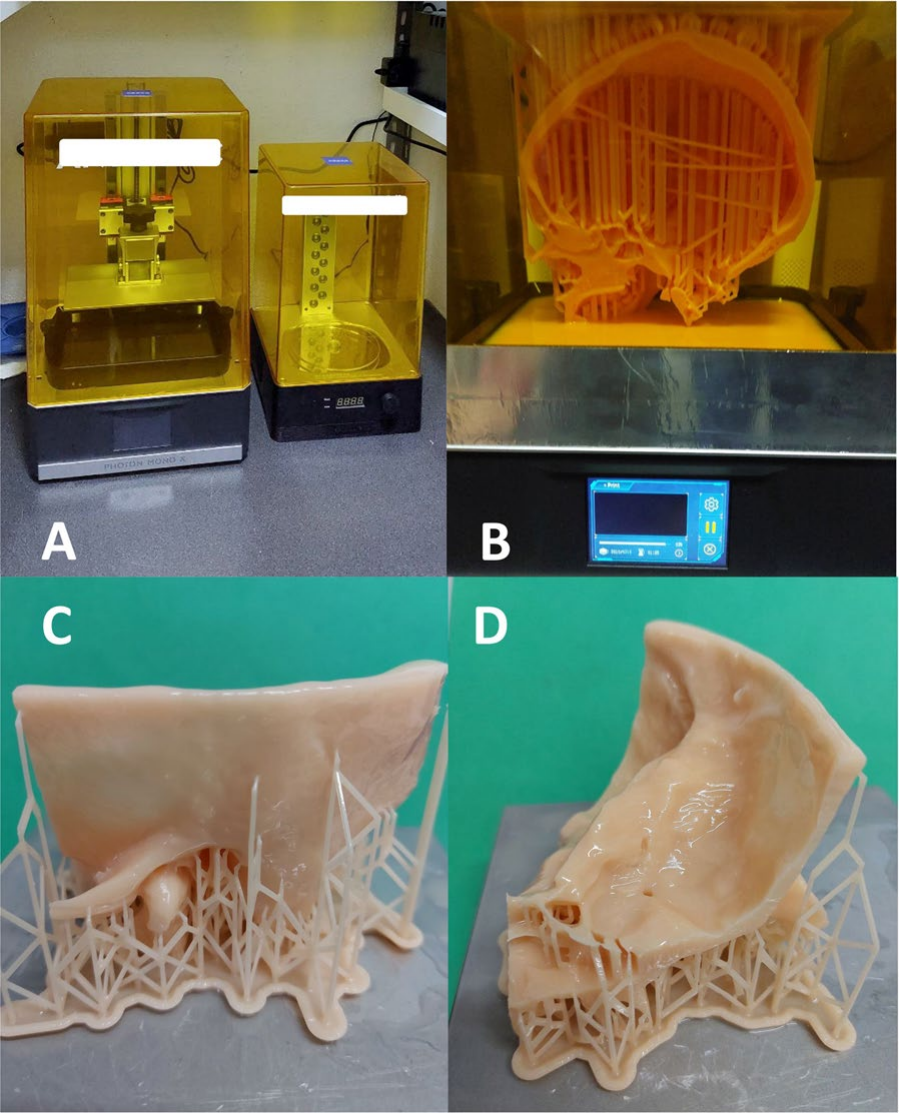
**3**D-printing process: **A** Photon mono × 4 k desktop printer used to produce the ‘SAPIENS’ temporal bone models. This is a photopolymers (SLA) light-curing printer. **B** 3D-temporal bone model printing using the Photopolymerization system. An UV laser precisely traces the layer's pattern, causing the liquid resin to solidify where the light hits, while the rest remains liquid. After solidification, the build platform descends slightly, allowing a new layer of liquid resin to be exposed. The process repeats, layer by layer, solidifying and stacking each new layer is added to the previous one until the entire object is formed. **C**, **D** Final phase of temporal bone realization. It is necessary to eliminate excess uncured resin from the surface and empty anatomical structures of the temporal bone, completing the curing process to create a usable TB model. Initially, the model is separated from the printing base with inserted printing supports

The printing program LycheeSlicer (2023 © Mango3D) was employed for the 3D printing. The Photon mono x 4 k desktop printer was used to produce the temporal bone models. This is a photopolymers (SLA) light-curing printer (Fig. 1A). The choice of an SLA printer was crucial to reconstruct anatomical structures with high detail.

Figure 1B shows the layer-by-layer SLA 3D printing. SLA printer can use different types of resin for the model printing. The difficulty of choosing the best material was due to the identification of a resin with physical characteristics similar to the human temporal bone. Considering that SLA resins do not undergo plastic deformation, flexural strength and elasticity were evaluated, in order to select a material that could offer human bone-like sensations and responses during drilling. The resin was therefore chosen comparing the parameters of various materials with the cortical bone and making different test models with various resins that were subsequently dissected. Anycubic standard resin was finally selected to produce temporal bone printed models.

### Post-printing phase

**Fig. 2:**
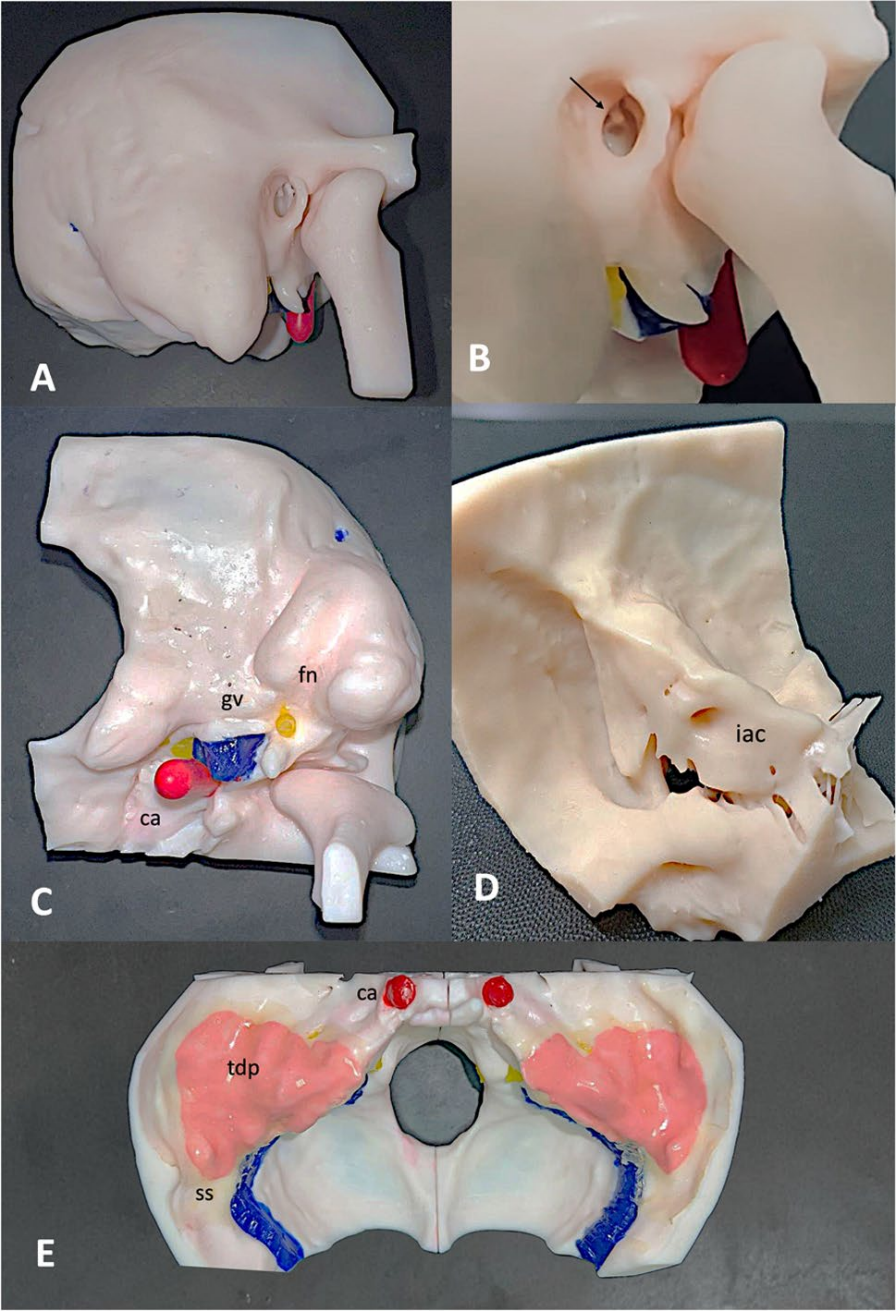
Final 3D-printed ‘SAPIENS’ temporal bone model. **A** Lateral coronal view; external auditory canal, mastoid bone, stylomastoid process and zygomatic process are visible. **B** magnified lateral coronal view; arrow indicates incudo-stapedial joint and stapes superstructures visible from the EAC. **C** External skull base and temporal bone axial view; the emergence from the skull base of the facial nerve (fn) marked in yellow, the carotid artery (ca) marked in red and the jugular vein (gv) marked in blue are visible. **D** lateral and view of the internal skull base and petrous bone. Internal auditory canal (iac) is visible. **E** Axial view of the internal skull base and petrous bone. The temporal bone plate has been marked with a pink color and a layer of soft tissue to simulate the dura of the middle cranial fossa during dissection was placed. Sigmoid sinus (SS) and carotid artery are visible (ca)

The post-printing phase is crucial for achieving high-quality manufacturing. It is necessary to eliminate excess uncured resin from the surface and empty anatomical structures of the temporal bone, completing the curing process to create a usable TB model. Initially, the model is separated from the printing base with inserted printing supports Care is taken to remove the supports without applying direct force to the anatomical structures (Fig. 2C, D). Due to the anatomical complexity of the temporal bone, which consists of hidden empty structures, a thorough cleansing process is conducted. The anatomical structures are cleaned by injecting isopropyl alcohol with a syringe. The facial nerve is irrigated from the stylomastoid foramen, ensuring the internal surface of the facial canal is cleansed. Injecting the solvent into the internal acoustic meatus allows for the cleaning of the semicircular canals from any residues, and excess liquid is drained from the oval window. The prepared model is then placed in a basket, inserted into a magnetic agitator containing isopropyl alcohol, to thoroughly clean the surface, leaving it to wash for 15 min. This duration is necessary due to the complex morphology of the structure; a shorter time might not ensure proper cleansing. For the drying phase, everything is left to dry for 3 h, allowing excess alcohol solution to come out of the model's cavities. The final phase of the model involves the completion of polymerization in a light-curing machine specially designed to evenly distribute light at each point.

The final step in the production of our 3D-temporal bone model includes creating the soft structures within the temporal bone to closely mimic the human temporal bone. The facial nerve, internal carotid, transverse sinus, sigmoid sinus, jugular gulf, and facial nerve are reproduced using paints and silicones of different colors and compositions.

### Final characteristics of the 3D-printed temporal model

The 3D-printed temporal bone model measured approximately 20 cm in length, 18 cm in width, and had a circumference of 50–60 cm. It weighed 75 g, with a production cost of about €40 per model. Printing and post-printing processes took three hours and one hour, respectively, for a total of 4 h required to produce each single model.

### 3D-printed temporal bone model dissection and validation

Five ENT surgeons, each possessing extensive and certified experience in otologic surgery, were invited to perform the dissection of the final 3D-printed TB model. Among these surgeon raters, four were board-certified otologists with post-residence experience of 12, 10, 8, and 3 years, respectively. The fifth rater was a neuro-otologist with 30 years of experience in this surgical domain. To prevent conflicts of interest, these surgeons were carefully selected from those who did not belong to the research group that designed and developed the 3D-printed model.

The surgeons conducted the 3D-printed TB dissection in the temporal bone laboratory of Sapienza University of Rome, using standard operating theater equipment (microscope, micro-drills, irrigation-suction system) and all necessary personal protective equipment (gowns, gloves, masks, and eye protection).

After performing the 3D-printed TB model dissection, each surgeon completed a questionnaire assessing their experience with the model and how well it approximated a human cadaveric temporal bone. We chose to utilize a specific questionnaire developed by Mowry et al. to evaluate 3D-temporal bone models. This questionnaire had been previously employed in a multi-institutional study comparing various TB models and in other clinical studies evaluating new 3D-printed temporal bone models. The questionnaire comprised 15 queries investigating characteristics of the 3D-printed TB Model, middle ear and mastoid anatomy, and the reproducibility of different surgical dissection steps. Fourteen questions were presented on a 4-point Likert scale (1—being far from reality, to 4—very real). The final question inquired if the model could genuinely be used as a simulator for surgical practice (utilizing a 5-point scale in this case). The maximum score allowed was 61, and a total score exceeding 40 was considered indicative of a 3D-printed model very similar to the human TB for anatomy and dissection.

Each surgeon was then asked to provide their general impressions of the 3D-printed model. The surgeon's input served as the reference to describe the characteristics of the 3D model in the results section.

## Results

### The ‘SAPIENS’ 3D-printed temporal bone model

**Fig. 3:**
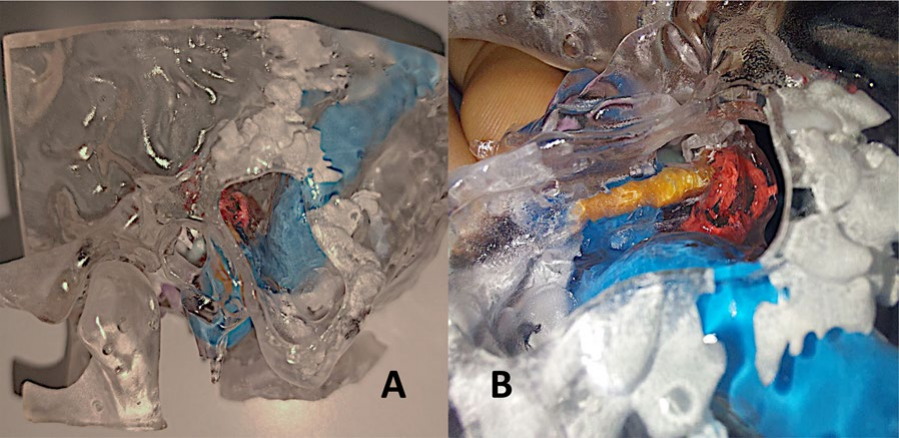
Transparent 3D-printed ‘SAPIENS’ temporal bone model; Transparent model created using colorless resin to the temporal bone and different-colored silicones to mark the inner ear structures. Cochlea and semicircular canals (red color), sigmoid sinus and jugular bulb (blu color), and facial nerve (yellow color). **A** Lateral coronal view, mastoidectomy has been performed in order to emphasize marked structures; **B** magnified lateral coronal view. Mastoidectomy performed. Facial nerve could be seen in its tympano-mastoid course

The final 'SAPIENS' 3D-printed model of the temporal bone is depicted in Fig. 2 A-E, demonstrating remarkable similarity to the human temporal bone in middle and inner ear anatomy. Figure 3A, B presents a transparent model created using colorless resin, realized to emphasize the accurate representation of inner ear structures (cochlea, semicircular canals, vestibule) and lateral skull base components (tegmen tympani, carotid artery, sigmoid sinus, and jugular bulb), well visible through injections of different-colored silicones.

**Fig. 4:**
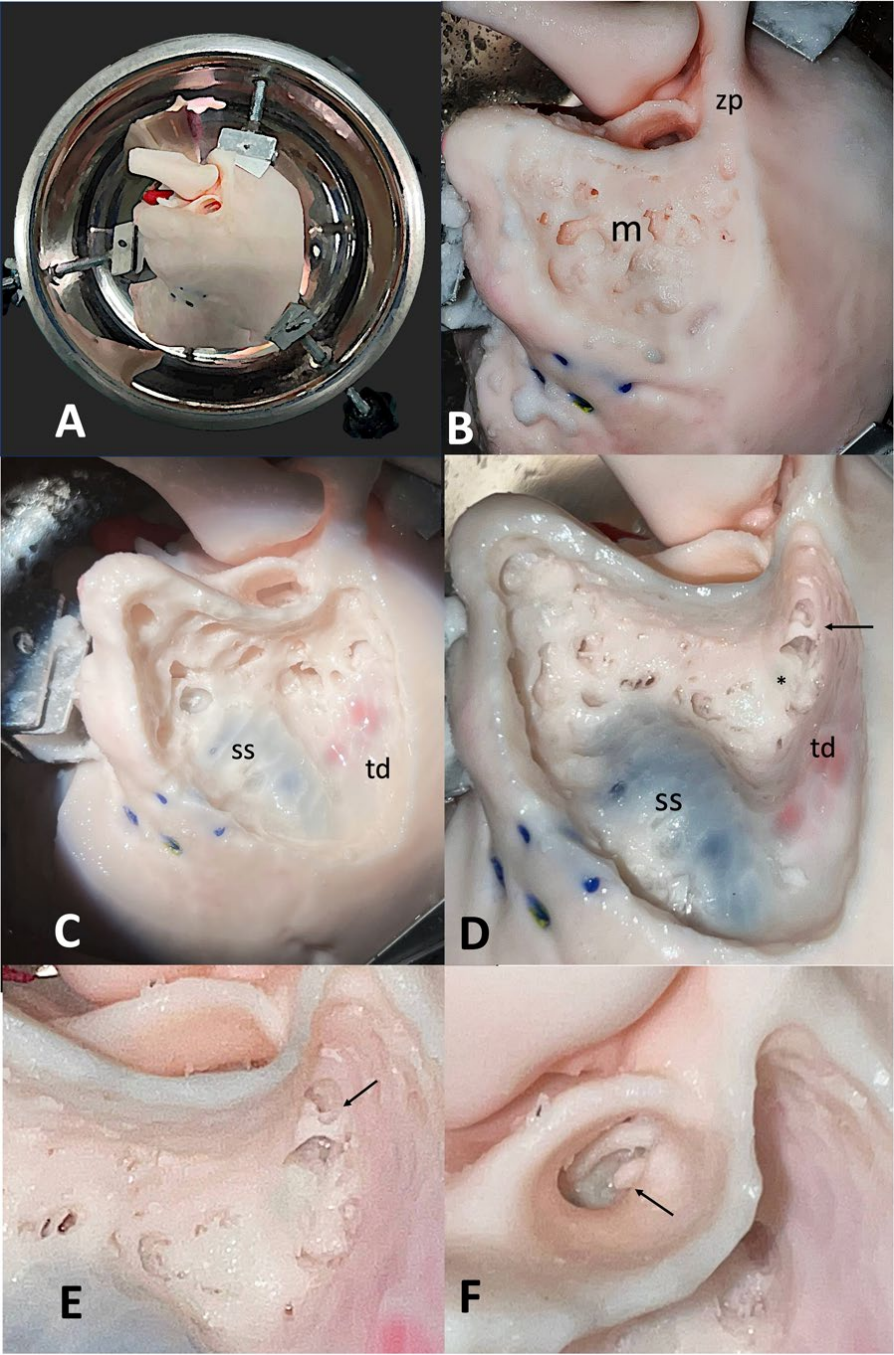
Mastoid dissection of the 3D-printed ‘SAPIENS’ temporal bone model (PART 1)—left temporal bone. Dissection was performed by a young surgeon in training (ENT residents) to demonstrate the effective use of this 3D-printed petrous bone model in onto-surgery training. **A** Temporal bone dissection setting using a specific temporal bone holder. **B** Cortical mastoidectomy a with well-represented mastoid bone pneumatization. Mastoid (m) zygomatic process (zp). **C** Mastoidectomy with sigmoid sinus (ss) and temporal dura plate (td) identification. **D** Mastoidectomy with epitympanotomy. Lateral semicircular canal (*) identification. The short process of the incus and the incudo-malleolar joint are visible (arrow). **E** mastoidectomy with epitympanotomy in a magnified view. The body of the incus and the head of the malleus with their ligaments are visible (arrow). **F** External auditory canal view; the long process of the malleus and the incudo-stapedial joint are visible (arrow)

**Fig. 5:**
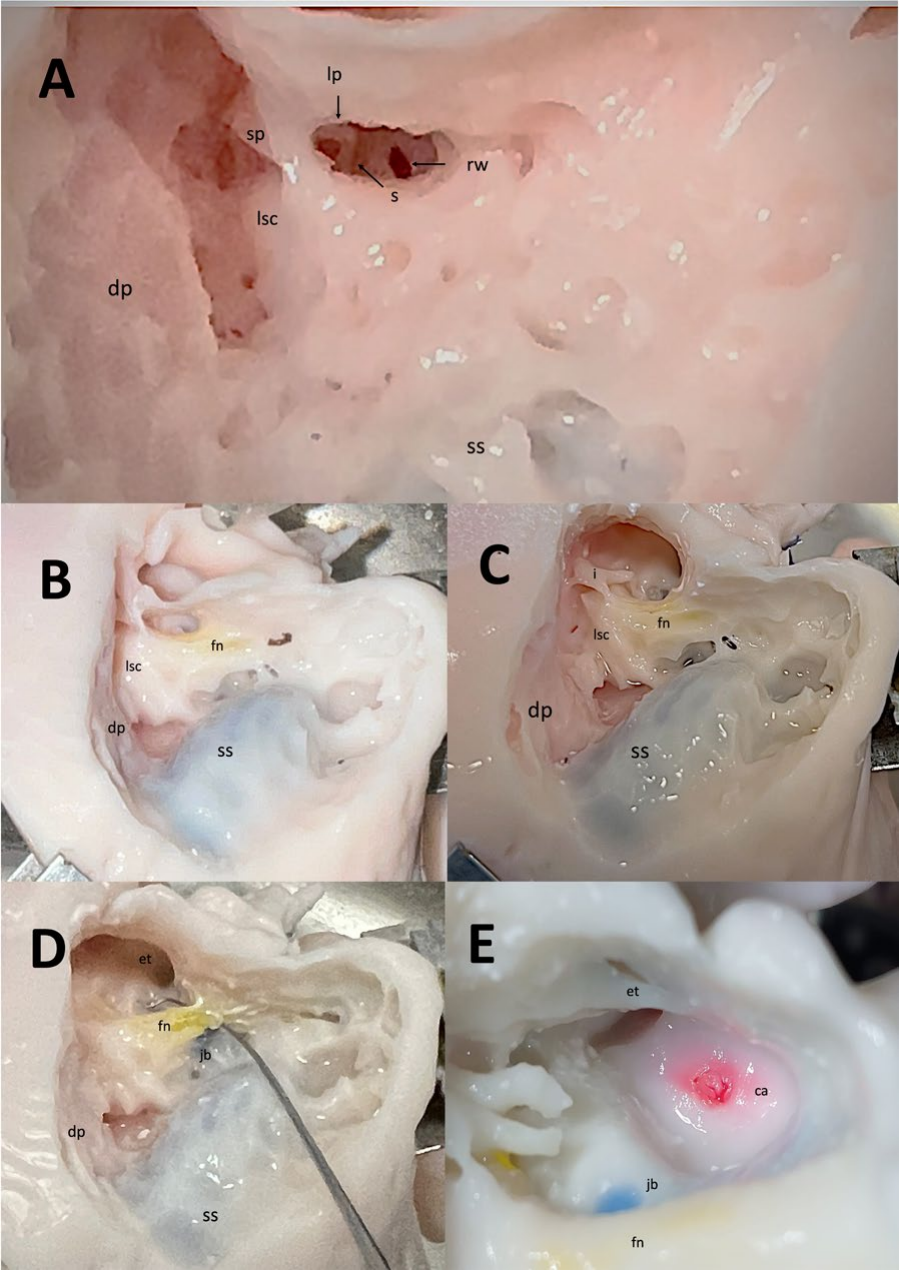
Mastoid dissection of the 3D-printed ‘SAPIENS’ temporal bone model (PART 1)—right temporal bone; Dissection was performed by a young surgeon in training (ENT residents) to demonstrate the effective use of this 3D-printed petrous bone model in onto-surgery training. **A** posterior tympanotomy; short process (sp) and long process (lp) of the incus, stapes (s), lateral semicircular canal (lsc) and round window (rw) are visible. **B** Facial nerve (fn) identified and scheletrized in its mastoid segment. **C** Removal of the posterior part of the external auditory canal. Malleus has been removed. Promontory, Eustachian tube orifice and incudo-stapedial joint are visible. **D** Scheletrized the facial nerve and dissection of the under-facial recess with access to the hypotympanic space and jugular bulb identification. **E** Identification of the internal carotid artery; jugular bulb (gb) and Eustachian tube (et) visible

Middle ear and mastoid dissection of the ‘SAPIENS’ 3D-temporal bone is reported in Figs. 4A–F and 5A–E. This dissection was performed by a young surgeon in training (ENT residents) to demonstrate the effective use of this 3D-printed petrous bone model in onto-surgery training.

### 3D-temporal model—questionnaire assessment

The average total score on the Mowry questionnaire was calculated as 49.4 ± 1.8, indicative of a 3D-printed model closely resembling the human TB in terms of anatomy and dissection. Sub-scores, based on responses from different surgeons, are reported in Table 1. Notable scores were achieved in the external contour (Q-1 average value of 4/4), sigmoid sinus contour (Q-6 average value of 3.2/4), cortical mastoidectomy simulation (Q-14 average value of 4/4), and the use of the model for simulating surgical practice (Q-15 average value of 4/4). Conversely, lower average values were reported for the texture of the drilled plastic mastoid architecture (Q-2 average value of 2.6/4), otic capsule contour and density (Q-5 average value 2.2/4), and facial recess (Q-13 average value 2.2/4). Importantly, no sub-queries reported a negative value below 2.

**Table 1:** Mowry questionnaire evaluating temporal bone dissection of the 3D-printed models

	Tester 1	Tester 2	Tester 3	Tester 4	Tester 5	Average sub-scores values
1. External contour	4	4	4	4	4	4
2. Texture of the drilled plastic mastoid architecture	3	3	2	3	2	2,6
3. Anatomy of the antrum (incus pointer)	3	3	3	3	3	3
4. Tegmen contour	3	3	3	3	2	2,8
5. Otic capsule contour and density	2	2	2	2	3	2,2
6. Sigmoid sinus contour	3	3	3	3	4	3,2
7. Did the model recreate the change in pitch heard during in vivo temporal bone surgery?	3	2	3	3	4	3
8. How is the reflectivity of the model? (how much the light penetrated the material to allow visualization of the deeper structures)	3	4	3	4	3	3,4
9. Absence of odor production when dissected	4	4	4	4	4	4
10. Dust formation during dissection	4	3	4	3	4	3,6
11. Supporting material absence	4	3	3	4	4	3,6
12. Facial nerve	3	2	3	3	3	2,8
13. Facial nerve recess	3	2	3	2	3	2,6
14. Overall, do you feel the best bone accurately recreated a model for cortical mastoidectomy?	4	4	4	4	4	4
15. Do you feel this model can be used as a simulator for surgical practice?	5	5	4	5	4	4,6
Total values	51	47	48	50	51	

### 3D-temporal model general evaluation

The 3D model was consistently deemed very similar to the human temporal bone in dissection practice. The mastoid displayed well-pneumatized and compact bone coverage. The texture of the mastoid architecture, mimicking that of a human temporal bone from a cadaver, facilitated realistic drilling. The sigmoid sinus, marked with a blue dye, was distinctly visible during mastoidectomy. The tegmen tympani, highlighted with a pink dye, remained recognizable in transparency during mastoid dissection, providing accurate guidance on the direction of the dura. The ossicular chain, round window, promontory, and Fallopian canal with the facial nerve injected with silicone (yellow color) were well represented and dissectable. The internal carotid artery (stained red) and jugular bulb (stained blue) were easily identified during dissection. The semicircular canals, vestibule, and cochlea, exhibiting increased resin consistency to simulate a more compact labyrinthine bone, appeared empty during the dissection process. Notably, the model did not exhibit abnormal fracturing during dissection and provided a tactile feel, sound, and visual appearance akin to cadaveric temporal bones. All surgeons unanimously considered this model an excellent choice for educational purposes and skill enhancement in young otological surgeons in training.

## Discussion

Surgical simulation is widely recognized as an important tool in surgical education, avoiding unskilled surgeons and reducing medical errors [15–21]. Ideally, clinical simulations, as a part of training, allow for the learning and development of skills in a realistic yet safe environment. Unfortunately, simulating otologic surgery is not easy. Cadaveric temporal bone dissection remains the gold standard for otologic simulation training, but it should not be forgotten that this precious resource presents some disadvantages and limitations. Access to cadaveric temporal bones is limited and depends on the availability of cadaver donors. Fresh cadaveric specimens are preferred for otologic dissection to better mimic surgical conditions, but access to these fresh cadavers can be even more limited and logistically challenging. Cadaveric TB specimens also deteriorate over time and need good preservation methods, limiting the duration and utility of training sessions and research projects [17–25]. The use of formaldehyde and other preservation chemicals in cadaveric specimens can have environmental and health-related concerns due to the biological risk of these specimens. Finally, the use of human cadavers for educational and research purposes involves ethical and legal considerations, including consent, privacy, and proper handling and disposal [21, 22].

Due to all these limitations of human TB from cadaver, in recent years, there has been a growing interest in complementary or alternative methods for otologic training and research, such as 3D-printed anatomical models, virtual reality simulations, and live-tissue training models. These approaches aim to address some of the limitations associated with cadaveric temporal bone dissection and provide additional opportunities for skill development and research in otology [15–30].

Three-dimensional printing of temporal bones is an enabling innovation and advancement in the field of innovative surgical education methods. Different investigators have independently developed 3D-printed temporal bone models for use in surgical training as well as for the preoperative simulation of challenging operative cases [13–30]. These detailed temporal bone models are produced with additive manufacturing 3D slicers processes, which is a creative process of 3D objects by adding successive layers of material in different shapes. Air spaces within and surrounding an object often require the use of support material, a water-soluble gel that is easily removed after printing, thus creating empty cavities with the structures in the center (middle ear and ossicular chain, Fallopian canal). Therefore, 3D printing is efficient in the creation of anatomic models with highly detailed internal anatomy, such as the temporal bone. Different materials (thermoplastic or light-curing resins) and printing methods have been developed and proposed, each showing different characteristics of the final model and synthetic bone drilling [13–30].

In 2010, Bakhos et al. [26] used white resin to reproduce the bony anatomy of the temporal bone based on CT scans of cadaveric specimens. Ossicular detail was sufficient for the simulation of middle ear prosthesis placement in 2 of the models. In 2013, Mick et al. [27] developed a temporal bone prototype with an improved similarity to bone, and in multiple colors, using plaster powder and a binding agent containing cyanoacrylate. The middle fossa plate was coated with latex paint to simulate dura. In 2015 Mowry et al. [16] produced a plastic simulated temporal bone on an inexpensive 3D printer that showed the visual and haptic experience of drilling similar to a human temporal bone. A group of expert otologic surgeons evaluated this temporal bone model using the first version of the questionnaire reported in this study. Raters felt that the printed model accurately represented the external TB contour, sigmoid sinus, tegmen, and antrum. Two raters gave the fragmentation of the plastic by the drill poor scores. Despite lower scores in facial anatomy and along the otic capsule, raters reported the model was acceptable as a training device for cortical mastoidectomy. They felt it could be used as a simulator for planned surgery and accurately reproduced a model for cortical mastoidectomy.

Recently, Gadaleta et al. [18] developed a very realistic temporal bone model that was deemed appropriate in surgical train dissection by 10 ENT residents in training. Participants found this 3D model to be like cadaveric temporal bones, particularly in safety, overall anatomy, and mastoid dissection. The total cost of the material required to fabricate the model was low (approximately $1.50). The main limitation of the specimen was considered the facial nerve canal and ossicular chain were not segmented and included in the models.

In 2021 the American Academy of Otolaryngology–Head and Neck Surgery Foundation’s (AAO-HNSF’s) 3D-Printed Temporal Bone Working Group was formed with the goal of sharing information and experience relating to the development of 3D-printed temporal bone models. The group conducted a multi-institutional study to directly evaluate and compare 12 3D-printed TB models. The models evaluated in this study demonstrated significant anatomic detail and a likeness to human cadaver specimens for drilling and dissection. Besides, models printed in standard resin material with a stereolithography printer scored the highest values of dissection feedback, though the margin of difference was negligible in several categories [15].

In this original article, we describe our project of a 3D-printed temporal bone developed for educational, training programs, and improving surgical skills in the ENT Department of Sapienza University of Rome.

The evaluative dissection of our model was entrusted to five surgeons with expertise in otological surgery, who were not involved in the implementation of the project. To assess our model, each surgeon answered the questionnaire developed by Mowry et al. The average value of the total score of our model was calculated as 49.4 ± 1.8, which, according to the results of Mowry et al., was indicative of a 3D-printed model very similar to the human TB for anatomy and dissection. The value of our model appeared to be higher than that reported for the models analyzed in the study of Mowry et al., in which the best three models reported overall values of 46, 45.33, and 44.33, respectively.

Analyzing the questionnaire sub-score values, it emerged that the strong points of our model were the external contour (average value of 4/4), Sigmoid sinus contour (average value of 3.2/4), cortical mastoidectomy simulation (average value of 4/4), and use of the model to simulate surgical practice (average value of 4/4). Conversely, lower average values were reported in the texture of the drilled plastic mastoid architecture (2.6/4), Otic capsule contour and density (Q-5 average value 2.2/4), and facial recess (Q-13 average value 2.2/4).

According to the general comments of otologic surgeons testing our model, it was considered useful to improve surgical skills in young surgeons in training. The anatomy of the middle ear and facial nerve was faithfully reproduced. Mastoidectomy, epitympanotomy, posterior tympanotomy, and canal wall down technique were well simulated. Marked structures (sigmoid sinus, tegmen tympani, and facial nerve) made the dissection more realistic and similar to real otological surgery.

Further studies are in progress to test the use of the SAPIENS 3D-temporal bone model in shorten the learning curve of young oto-surgeons.

According to our experience and literature review, we can state that 3D-printed temporal bone models offer several advantages and disadvantages in the fields of education and surgical otological training.

*Definite advantages can be considered*:

- *Improved visualization.* 3D printing allows for the creation of highly detailed and accurate anatomical models, providing a better understanding of the temporal bone's complex structure.
- *Hands-on learning.* Medical students, residents, and surgeons can use 3D-printed temporal bone models to gain practical experience and enhance their surgical skills without the need for cadaveric specimens.
- *Preoperative planning.* Surgeons can use 3D-printed temporal bone models to plan and simulate complex surgeries, such as cochlear implantation or mastoidectomy. This helps optimize surgical outcomes and reduces the risk of complications.
- *Customization.* 3D printing allows for the creation of patient-specific models based on medical imaging data, enabling surgeons to practice on replicas that closely resemble the actual patient's anatomy. Also, temporal models can be created with middle ear pathologies rarely found in cadaveric bones.
- *Reduced cost.* While initial setup costs for 3D printing may be high, it can ultimately reduce costs in medical training and research by eliminating the need for disposable models or cadaveric specimens.
- *Reproducibility* Once a 3D model is designed and validated, it can be reproduced consistently, ensuring that multiple learners or researchers have access to the same high-quality learning or research tool.
- *Implants testing.* Researchers and clinicians can use 3D-printed temporal bone models for medical device tests, such as hearing aids or implants, without harming the patient.
- *Patient education.* 3D models can be used to educate patients about their conditions and planned surgical procedures, improving patient understanding and informed consent.
- *Research.* Researchers can use 3D-printed temporal bone models to study various aspects of the ear, conduct experiments, and test new medical devices and treatments.

*3D printing of temporal bone also shows some disadvantages and challenges associated with this technology*:

- *Cost.* 3D printing can be expensive, particularly when using high-quality materials and printers. The initial investment in equipment and materials may be prohibitive for some institutions or individuals.
- *Complexity.* Creating accurate and anatomically faithful 3D models of the temporal bone can be technically challenging. It requires expertise in medical imaging, 3D modeling software, and knowledge of the specific anatomy.
- *Time-consuming.* The process of designing, printing, and post-processing 3D models can be time-consuming. It may not be suitable for urgent medical situations or research projects with tight deadlines.
- *Quality control.* Ensuring the accuracy and fidelity of 3D-printed models is crucial. Small errors or imperfections in the model can lead to incorrect educational or surgical outcomes.
- *Material limitations.* The choice of printing material can impact the realism of the model. While some materials closely mimic bone properties, others may not offer the same level of detail or durability.
- *Limited haptic feedback.* While 3D-printed models provide visual and tactile feedback, they may not fully replicate the sensation of real tissue or bone, which can be important for surgical training.
- *Environmental impact.* The production of 3D-printed models consumes energy and resources. Additionally, the disposal of waste materials generated during the printing process can have environmental implications. Some models also need longer printing times that could be > 5 h for each model.
- *Lack of standardization.* There is currently no universally accepted standard for creating and evaluating 3D-printed anatomical models. This lack of standardization can lead to variability in model quality and accuracy.

We recognize that several challenges must be addressed in the development of an optimal temporal bone simulator, one that can compare favorably with cadaver specimens or actual surgeries in the operating room. Of paramount importance is the ability to accurately reproduce the highly detailed bony and soft tissue anatomy characteristic of the human temporal bone. An additional challenge involves the selection of materials capable of faithfully recreating not only the sensation of drilling through cortical and trabecular mastoid bone, but also the dense otic capsule bone.

These models must also facilitate the identification and dissection of critical soft tissue structures, including the cochlea, labyrinth, facial nerve, sigmoid sinus, dural plates, and internal carotid artery, while effectively replicating the complex nerve and vascular anatomy of the lateral skull base [15–27].

In our SAPIENS temporal bone model, all structures of the inner ear such as the cochlea, semicircular canals and facial nerve in the labyrinthine segment have been reconstructed and marked. Further studies are in progress to test our 3D model in labyrinthine and cochlear dissection and internal auditory canal access.

A forthcoming challenge will be to utilize preoperative CT scans as a basis for 3D printing temporal bone models that mimic individual pathological clinical cases, such as malformations or complex anatomies. This approach will enable surgeons to practice the dissection of artificial models before performing similar surgeries on patients, thereby reducing medical errors and enhancing surgical performance.

Finally, different 3D-printed models have been developed in different countries [15–30], however, few models have been commercialized. Comparative studies between our model and commercialized 3D-printed and temporal bone models are underway.

## Conclusion

In the coming years, innovative learning tools will serve as valuable adjuncts alongside cadaveric teaching, which is currently considered the gold standard method for teaching anatomy. Along this path, we have designed and developed a 3D model of the temporal bone that closely resembles the human temporal bone. This model enables the surgical dissection of the middle ear and mastoid with an excellent degree of similarity to the dissection performed on cadaveric temporal bones. We anticipate that our model will be a useful resource for junior otological surgeons, aiding in the improvement of their anatomical knowledge and surgical skills. New studies are underway to enhance the quality of our model and bring it even closer to the tissue characteristics of the human anatomy.
